# NT5DC2 promotes leiomyosarcoma tumour cell growth via stabilizing unpalmitoylated TEAD4 and generating a positive feedback loop

**DOI:** 10.1111/jcmm.16409

**Published:** 2021-05-16

**Authors:** Bowen Hu, Shijie Zhou, Xuefeng Hu, Hua Zhang, Xiaorong Lan, Mei Li, Yunbing Wang, Qinsheng Hu

**Affiliations:** ^1^ Department of Orthopedics Orthopedics Research Institute West China Hospital Sichuan University Chengdu China; ^2^ Cancer Center West China Hospital Sichuan University Chengdu China; ^3^ National Engineering Research Center for Biomaterials Sichuan University Chengdu China; ^4^ Department of Head & Neck Cancer Cancer Center West China Hospital Sichuan University Chengdu China

**Keywords:** leiomyosarcoma, NT5DC2, TEAD4, ubiquitin‐proteasome pathway

## Abstract

5′‐Nucleotidase Domain Containing 2 (NT5DC2) is a novel oncoprotein, the regulatory effects of which have not been well characterized. This study aimed to investigate the expression profile and functional regulation of NT5DC2 and its potential interplay with TEAD4 in leiomyosarcoma (LMS). Bioinformatic analysis was conducted using data from The Cancer Genome Atlas (TCGA) and Genotype‐Tissue Expression (GTEx) program. LMS cell lines SK‐LMS‐1 and SK‐UT‐1 were used for both in vitro and in vivo analysis. Results showed that *NT5DC2* is aberrantly upregulated in LMS. Its overexpression was associated with unfavourable survival. Deletion of *NT5DC2* significantly reduced the expression of cyclin B1, cyclin A2, cyclin E1 and CDK1 and increased G1 phase arrest in LMS cell lines, and suppressed their proliferation both in vitro and in vivo. NT5DC2 interacted with unpalmitoylated TEAD4, and this association reduced TEAD4 degradation via the ubiquitin‐proteasome pathway. TRIM27 is a novel E3 ubiquitin ligase that induces K27/48‐linked ubiquitination of unpalmitoylated TEAD4 at Lys278. *TEAD4* inhibition significantly suppressed LMS cell growth both in vitro and in vivo. Dual‐luciferase assay demonstrated that TEAD4 could bind to the NT5DC2 promoter and activate its transcription. Based on these findings, we infer that the NT5DC2‐TEAD4 positive feedback loop plays an important role in LMS development and might serve as a potential therapeutic target.

## 1 INTRODUCTION

Leiomyosarcoma (LMS) is a rare cancer that derives from soft tissues such as smooth muscle, nerves, arteries and veins. LMS mainly includes uterine leiomyosarcoma (ULMS) and soft tissue leiomyosarcoma (STLMS), according to the histological origination. Some recent large studies tried to outline the genomic landscape of LMS.^1^, ^2^ They found that LMS has low somatic mutation burdens (limited to *TP53, ATRX*, and *RB1*), depletion of some critical tumour suppressor genes (such as *TP53, RB1*, *CDH1* and *PTEN*) and high expression of genes linked to myogenic differentiation (such as *MYLK, MYH11, ACTG2*, *miR‐143* and *miR‐145*) and PI3K‐AKT‐MTOR signalling (typically *IGF1R*, *AKT*, *RICTOR* and *MTOR*).^1^, ^2^ However, the molecular alterations at the post‐transcriptional level remain largely unclear.

5′‐Nucleotidase Domain Containing 2 (NT5DC2) is a protein with potential metal ion binding and 5′‐nucleotidase activity, according to Gene Ontology (GO) annotations. However, the exact molecular function is still not quite clear. Some recent studies revealed that this protein might have an important role in cancer biology. It enhances glioma stem‐like cell tumorsphere formation and cell viability in vitro and promotes tumorigenesis in vivo.^3^ Mechanistically, it binds to Fyn, an Src family proto‐oncogene, and suppresses its degradation.^3^ Its overexpression leads to increased proliferation and reduced apoptosis of non‐small‐cell lung cancer cells, via reducing p53 expression.^4^ It promotes the proliferation and colony formation of hepatocellular carcinoma (HCC) in vitro and facilitates tumour growth in vivo.^5^ Its physical interaction with the epidermal growth factor receptor (EGFR) directly reduces EGFR ubiquitination and degradation,^5^ which is a high potential therapeutic target in multiple cancers.^6^ These findings imply that NT5DC2 might exert critical oncogenic effects via acting as an oncoprotein stabilizer.

The TEAD family of transcription factors (TEAD1, TEAD2, TEAD3 and TEAD4) are the terminal nuclear effectors of the Hippo pathway,^7^ which is usually overactivated in sarcoma.^8^ TEAD activation plays critical roles in cancer biology, including epithelial‐to‐mesenchymal transition (EMT), proliferation, metastasis, chemoresistance and cancer stem cell properties via their transcriptional target genes.^7^ Recent studies revealed that post‐translational S‐palmitoylation of TEAD is important for its stability and activity.^9^, ^10^ After depalmitoylation by depalmitoylases (such as APT2 and ABHD17A), unpalmitoylated TEAD4 can be degraded via the ubiquitin‐mediated proteasome pathway.^11^ However, the regulatory mechanisms that dynamically control TEAD4 degradation and their functional role in LMS have not yet been identified. In this study, we aimed to investigate the expression profile and functional regulation of NT5DC2 and its potential interplay with unpalmitoylated TEAD4 in LMS.

## 2 MATERIALS AND METHODS

### 2.1 Data retrieved from The Cancer Genome Atlas (TCGA) and Genotype‐Tissue Expression (GTEx) program

Data extraction from TCGA and GTEx was performed using the UCSC Xena Browser,^12^ as we described previously.^13^ Log_2_ transformed transcript per million (TPM) (log_2_ (TPM + 0.001)) was used for gene expression and comparison. Progression‐free survival (PFS), disease‐specific survival (DSS) and disease‐free survival (DFS) were extracted for survival analysis. LMS subtype data were obtained from one previous publication of TCGA Research Network.^1^ 80 cases were diagnosed as LMS, including 27 ULMS and 53 STLMS. The integrated pathway level (IPL) for TEAD4, which was calculated by the PARADIGM (PAthway Recognition Algorithm using Data Integration on Genomic Models)^14^ in 80 LMS cases was extracted. In brief, this algorithm infers IPL scores for genes, complexes and processes by integrating diverse high‐throughput genomics information with known signalling pathways from a single cell line or patient sample.^14^

### 2.2 Immunohistochemistry (IHC)

NT5DC2 IHC in normal smooth muscle and HCC tissues were reviewed using data provided in the Human Protein Atlas,^15^ in which NT5DC2 was stained by HPA050683 (Sigma‐Aldrich).

LMS tissue array was purchased from Taibsbio. IHC staining of NT5DC2 was performed using anti‐NT5DC2 (HPA050683, Sigma‐Aldrich), according to the method introduced previously.^16^

### 2.3 Cell culture and treatment

LMS cell line SK‐LMS‐1 and SK‐UT‐1 were purchased from ATCC and were cultured in Dulbecco's modified Eagle's medium (DMEM) supplemented with 10% foetal bovine serum (FBS) (HyClone Laboratories), 100 U/mL penicillin, and 100 μg/mL streptomycin. The cell line was authenticated by matching the short‐tandem repeat (STR) DNA profiles according to the provider. Lentiviral shRNA targeting *NT5DC2* and *TEAD4* were constructed by HanBio Technology, with the shuttle plasmid pHBLV‐U6‐Puro. shRNA sequences used are provided in Table S1.

The genes encoding ubiquitin and its mutants with all lysine residues replaced by arginine ((K0), except K6 (K6^only^), K11 (K611^only^), K27 (K27^only^), K29 (K29^only^), K33 (K33^only^), K48 (K48^only^), or K63 (K63^only^) were synthesized (Sangon Biotech) and inserted into the shuttle plasmid pHBLV‐CMVIE‐IRES‐ZsGreen. Lentivirus expressing palmitoylation‐deficient (NM_003213.3, C335S, and C367A) *TEAD4* mutant with Flag tag (Flag‐mtTEAD4), Flag‐mtTEAD4 mutants (K73R, K118, K223R, K278R, and K282R), *NT5DC2* (NM_001134231.2) with Myc tag (Myc‐NT5DC2), TRIM27 (NM_006510.5) with Flag tag, TRIM54 (NM_032546.4) with Myc tag (Myc‐TRIM54), and STUB1 (NM_005861.4) with Myc tag (Myc‐STUB1) were also generated, based on the shuttle plasmid pLVX‐Puro. Lentiviruses used for infection were generated by co‐transfecting the packaging plasmids (pSPAX2 and pMD2.G) (HanBio Technology) in 293T cells, following the standard protocol recommended by the manufacturer. SK‐LMS‐1 and SK‐UT‐1 cells were infected with lentivirus at the multiplicity of infection (MOI) of 10. Proteasome inhibitor MG‐132 and protein synthesis inhibitor Cycloheximide (CHX) were purchased from Sigma‐Aldrich.

### 2.4 Reverse transcription‐quantitative PCR (RT‐qPCR)

Total RNA was extracted using RNeasy Mini Kit (QIAGEN). Then, the first‐strand cDNA was synthesized using a PrimeScript™ RT Kit (TaKaRa). qPCR reactions were conducted using an ABI 7900 HT thermocycler (Applied Biosystems), with SYBR Green Master Mix (Applied Biosystems). The threshold cycle (Ct value) of each sample was recorded and used to calculate relative gene expression using the 2^−ΔΔCt^ method. The primers used are provided in Table S1. *GAPDH* mRNA expression was used as an internal control.

### 2.5 Western blotting and co‐immunoprecipitation (co‐IP)

Total cellular protein was extracted using RIPA lysis buffer with protease inhibitor. 30 μg of the total protein was loaded to each lane, separated using 10% SDS‐PAGE, and electrotransferred onto polyvinylidene difluoride membranes (Millipore). Membranes were blocked, washed and then incubated with primary antibodies at 4℃ overnight. Then, all membranes were incubated with HRP‐conjugated secondary antibodies (Santa Cruz Biotechnology). The blots were then visualized with BeyoECL Star reagent (Beyotime) and an ImageQuant LAS‐4000 imaging system (GE Healthcare).

For co‐IP, the supernatant of cell lysate was collected after centrifugation and was precleaned by protein A/G PLUS‐Agarose (Santa Cruz Biotechnology). Then, the supernatants were immunoprecipitated with anti‐TEAD4 at 4°C for 6 hours under gentle agitation. After that, protein A/G PLUS‐Agarose beads was added to the mixture, with rotary agitation for 4 hours at 4°C. The immunoprecipitated complexes were collected, washed and subjected to western blot analysis. The input was used as a positive control.

Primary antibodies used included anti‐NT5DC2 (#PA5‐42853, ThermoFisher Scientific), anti‐TEAD4 (12418‐1‐AP, Proteintech), anti‐cyclin B1 (55004‐1‐AP, Proteintech), anti‐cyclin A2 (66391‐1‐Ig, Proteintech), anti‐cyclin E1 (11554‐1‐AP, Proteintech), anti‐CDK1 (19532‐1‐AP, Proteintech), anti‐HA (51064‐2‐AP, Proteintech), anti‐Myc tag (ab9106, Abcam), anti‐Flag tag (66008‐3‐IG, Proteintech), anti‐TRIM27 (12205‐1‐AP, Proteintech), anti‐TRIM54 (21074‐1‐AP, Proteintech), anti‐STUB1 (ab134064, Abcam) and anti‐β‐actin (66009‐1‐Ig, Proteintech).

### 2.6 CCK‐8 assay

CCK‐8 kit was used to detect cell viability according to the manufacturer's instruction. 5000 cells with or without lentiviral mediated gene suppression were seeded into each well of a 96‐well plate. Cell viability was measured at 24, 48, 72 and 96 hours after seeding. Absorbance at 450 nm was measured and recorded.

### 2.7 Colony formation assay

Colony formation was performed as previously described. Briefly, 1000 cells were seeded into a six‐well plate and cultured for two weeks. The cells were then fixed and stained with 0.1% crystal violet for 15 minutes. Image J software was used to quantify the colonies.

### 2.8 Flow cytometric assay

Cells infected with lentiviral shNT5DC2 were harvested 48 hours after infection. Then, cells were fixed in 70% ethanol for 1 hour and then stained with propidium iodide (PI) solution containing RNase A (Sigma‐Aldrich) for 30 minutes at room temperature in the dark. Cell‐cycle distribution was then analysed by the Cellometer Vision CBA image cytometer (Nexcelom).

### 2.9 Immunofluorescent staining

SK‐LMS‐1 cells grown on coverslips were rinsed, fixed with PBS containing 4% paraformaldehyde for 10 minutes at room temperature and then permeabilized with PBS containing 0.1% Triton X‐100 for 10 minutes. After washing with PBS, cells were blocked for 30 minutes with 1% BSA in PBS at room temperature and then were incubated with primary antibody, including mouse Flag tag (66008‐3‐IG, Proteintech) and rabbit anti‐Myc tag (ab9106 Abcam) or rabbit anti‐TRIM27 (12205‐1‐AP, Proteintech) overnight at 4℃. After that, the cells were washed and incubated with DAPI (Beyotime) for 5 minutes. Then, the coverslips were sealed. Fluorescence images were captured using an AxioImager Z1 ApoTome microscope system (Carl Zeiss).

### 2.10 Dual‐luciferase assay

Wild‐type (WT) *NT5DC2* promoter sequence (Genome = hg38; chr3‐:52535227‐52533736) and the corresponding mutant sequences with mutant TEAD4 binding sites were chemically synthesized and cloned into the sites between KpnI and HindIII in pGL3 basic vector (Promega). SK‐LMS‐1 and SK‐UT‐1 cells with or without lentiviral mediated TEAD4 inhibition were seeded in 24‐well plates at a density of 2 × 10^5^ cells per well. 24 hours later, cells were transfected with 1 μg of recombinant vectors carrying WT or mutant (MT) promoter sequences, using Lipofectamine 2000 (Invitrogen). 0.05 μg of pRL‐CMV vector was co‐transfected to normalize the transfection efficiency. After transfection, cells were further cultured for 24 hours. Then, cells were lysed to measure firefly luciferase and renilla luciferase activities, using a dual‐specific luciferase assay kit according to the manufacturer's instruction (#E1910, Promega), with a luminometer (Promega).

### 2.11 Animal studies

Animal experiments were approved by the West China Hospital, Sichuan University. 5–6 weeks old, female BALB/c nude mice were purchased from Vital River Laboratory Animal Technology Co., Ltd. The mice were housed in a pathogen‐free animal facility and randomly assigned to the experimental or control group (6 mice per group). SK‐LMS‐1 cells were infected with lentiviral shRNA or scrambled (scr.) control. 48 hours later, cells were selected in a culture containing puromycin (1 μg/mL) for another 24 hours. Then, 5 × 10^6^ cells were implanted subcutaneously injected into the flanks of nude mice. Mice were sacrificed at 28 days. The tumour tissues were excised and weighed.

### 2.12 Statistical analysis

Statistical analysis was conducted using GraphPad Prism 8.1.2 (GraphPad Inc). One‐way ANOVA with post hoc Tukey's multiple comparisons and Welch's unequal variance *t* test were used for multiple‐group and two‐group comparison, respectively. Kaplan‐Meier (K‐M) survival curves were generated for survival comparison. Log‐rank test was performed to analyse the difference. Pearson's or Spearman's correlation coefficient (*R*) was calculated to estimate correlation. *P* < .05 was considered statistically significant.

## 3 RESULTS

### 3.1 High *NT5DC2* expression is associated with poor survival of LMS

**FIGURE 1:**
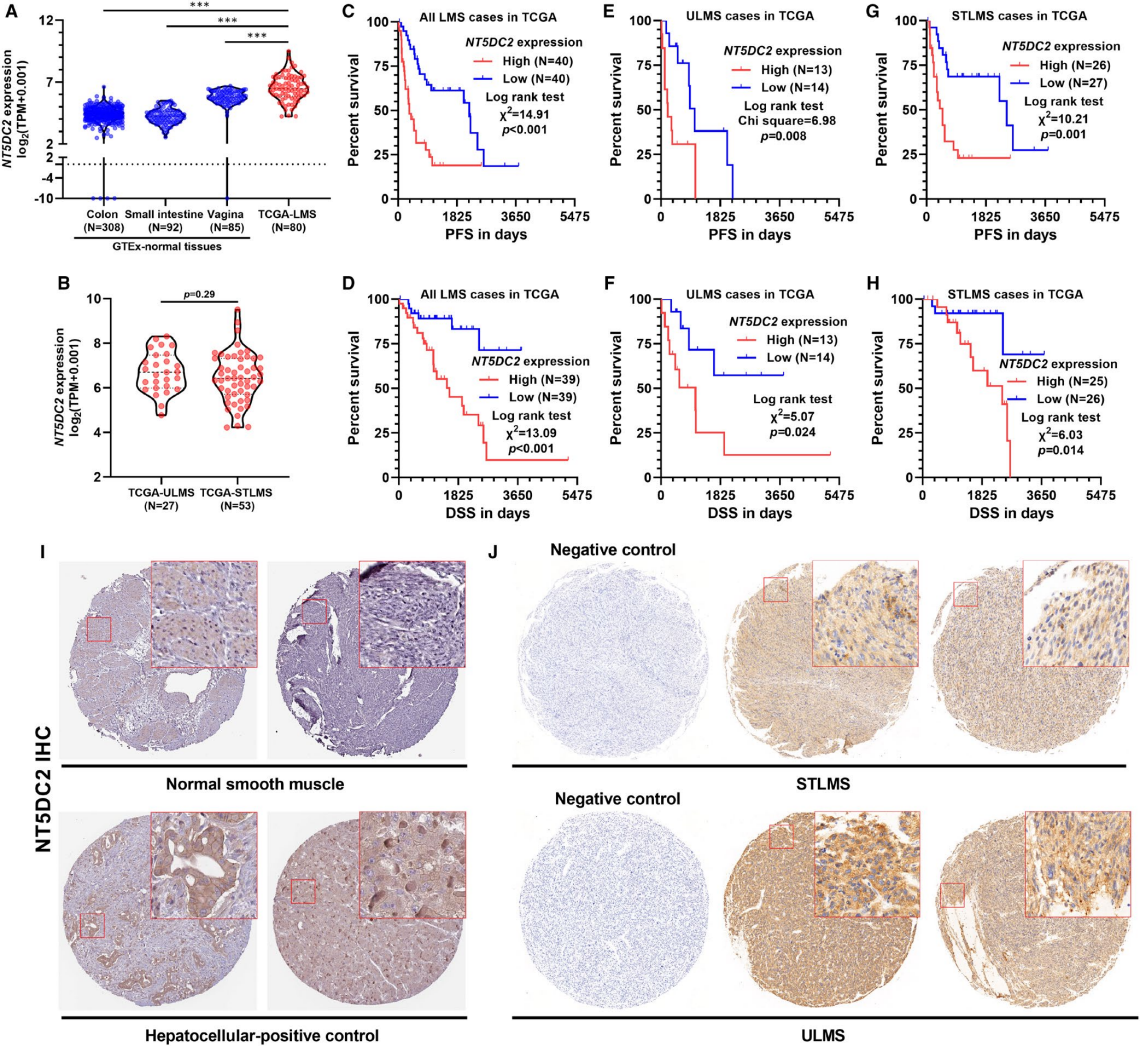
High *NT5DC2* expression is associated with poor survival of LMS. A, Comparison of *NT5DC2* expression in representative normal smooth muscle tissues, including colon (N = 308), small intestine (N = 92), and vagina (N = 85) from GTEx project and LMS tissues (N = 80). B, Subgroup comparison of *NT5DC2* expression between ULMS (N = 27) and STLMS (N = 53) cases in TCGA‐LMS. C‐H, K‐M survival analysis of PFS (C, E, and G) and DSS (D, F and H) in 80 LMS cases (C,D) and subgroup analysis in ULMS (E,F) and STLMS (G,H), respectively. I and J, IHC staining of NT5DC2 in normal smooth tissue (I, up), HCC tissue (I, down) and LMS tissues (J). Images of smooth and HCC tissues were obtained from the HPA, with the accesses: https://www.proteinatlas.org/ENSG00000168268‐NT5DC2/tissue/smooth+muscle and https://www.proteinatlas.org/ENSG00000168268‐NT5DC2/pathology/liver+cancer#img. *P < .05, **P < .01, ***P < .001

To characterize *NT5DC2* expression, we compared RNA‐seq data from three representative soft muscle tissues (colon, small intestine, and vagina) from GTEx and LMS tissue from TCGA‐SARC. Results showed that LMS group had significantly higher *NT5DC2* expression than the three normal groups (*P* < .001, Figure 1A). TCGA Research Network indicated that ULMS and STLMS had significantly different mRNA expression signatures.^1^ However, no significant difference was observed in *NT5DC2* expression between the two subgroups (*P* = .29, Figure 1B). Then, we performed K‐M survival analysis in all LMS cases and subgroups, respectively. Results showed that LMS cases with high *NT5DC2* expression had significantly worse PFS, DSS and OS than the counterparts with low *NT5DC2* expression (Figure 1C,D). Subgroup analysis confirmed the consistent trends in ULMS and STLMS (Figure 1E‐H). By retrieving IHC staining data in the HPA, we found normal smooth muscle tissue has low expression of NT5DC2 protein (Figure 1I, up). NT5DC2 expression was confirmed in HCC (Figure 1I, down), as reported previously.^5^ Using the same antibody in the HPA, we confirmed that both ULMS and STLMS cases had *NT5DC2* expression at the protein level, mainly in the cytoplasmic part of tumour cells (Figure 1J).

### 3.2 NT5DC2 promotes LMS cell‐cycle progression and proliferation in vitro and tumour growth in vivo

**FIGURE 2:**
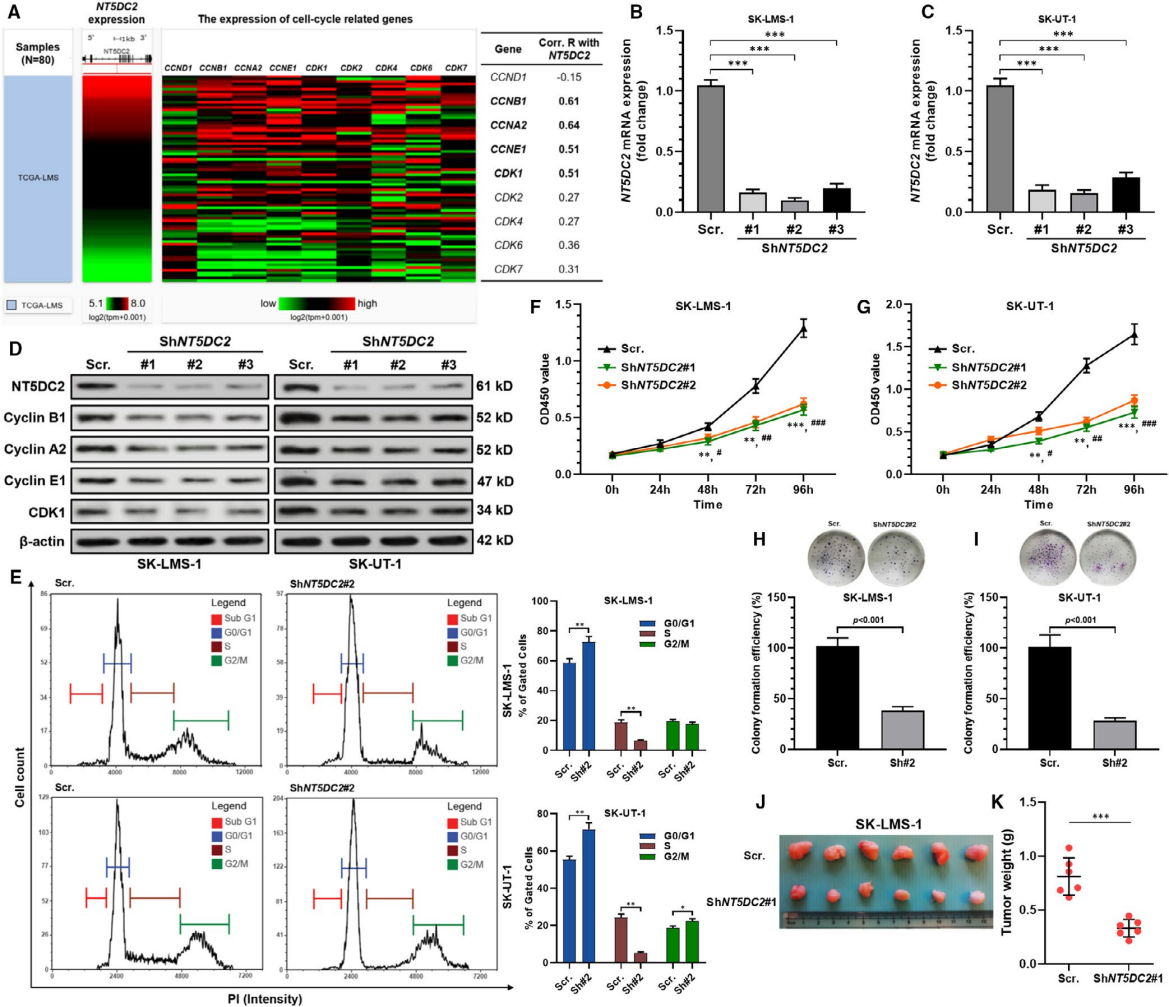
NT5DC2 promotes LMS cell‐cycle progression and proliferation in vitro and tumour growth in vivo. A, A heatmap (left) and correlation R chart (right) showing the correlation of NT5DC2 with multiple cell‐cycle related genes, including *CCND1, CCNB1, CCNA2, CCNE1, CDK1, CDK2, CDK4, CDK6* and *CDK7* in 80 LMS cases in TCGA. B and C, RT‐qPCR analysis showing the relative *NT5DC2* expression in ST‐LMS‐1 (B) and SK‐UT‐1 (C) cells 48 h after infection of lentivirus carrying *NT5DC2* shRNA or scramble control. D, Western blot assay of NT5DC2, cyclin B1, cyclin A2, cyclin E1 and CDK1 expression in ST‐LMS‐1 and SK‐UT‐1 cells 48 h after infection of lentivirus carrying *NT5DC2* shRNA or scramble control. E, 48 h lentiviral infection for *NT5DC2* shRNA or scramble control. SK‐LMS‐1 and SK‐UT‐1 cells were subjected to PI staining and flow cytometric analysis. Representative images of flow cytometric analysis of cell‐cycle distribution (left) and quantitation of cells in different phases (right) were given. F and G, The proliferation of SK‐LMS‐1 (F) and ST‐UT‐1 (G) cells with or without *NT5DC2* knockdown. H and I, Representative images of colony formation (up) and quantitation or relative colony capability (down) of SK‐LMS‐1 (H) and SK‐UT‐1 (I) cells with or without *NT5DC2* knockdown. J and K, SK‐LMS‐1 cells infected with *NT5DC2* knockdown (shRNA) or scramble control were subcutaneously implanted into nude mice, and the tumours were harvested 4 wk later (J). Compared with the controls, *NT5DC2* knockdown significantly inhibited tumour growth (K). *Comparison with sh*NT5DC2*#1, ^#^Comparison with sh*NT5DC2*#2, * and ^#^ *P* < .05, ** and ^##^ *P* < .01, *** and ^###^ *P* < .001

Previous studies imply that NT5DC2 enhances cell proliferation of multiple cancers.^5^, ^17^ Therefore, we hypothesized that NT5DC2 might also play an important role in LMS cell proliferation. By checking the correlation between *NT5DC2* and multiple cell‐cycle related genes, we found strong correlations (*R* ≥ .6) with *CCNB1* and *CCNA2* and moderate correlations (.6 > *R *≥ .4) with *CCNE1* and *CDK1* (Figure 2A). To validate the potential cell‐cycle regulation of NT5DC2, SK‐LMS‐1 and SK‐UT‐1 cells were transiently infected with lentiviral‐sh*NT5DC2* (Figure 2B,C). *NT5DC2* inhibition significantly decreased the expression of cyclin B1, cyclin A2, cyclin E1 and CDK1 at the protein level in the two cell lines (Figure 2D). Flow cytometric assay showed that after *NT5DC2* knockdown, both SK‐LMS‐1 and SK‐UT‐1 cells had increased G0/G1 arrest and reduced cells in the S phase (Figure 2E). G2/M arrest was also observed in SK‐UT‐1 cells (Figure 2E). CCK‐8 and colony formation assay confirmed that *NT5DC2* inhibition decreased the proliferation and colony‐forming ability of the two cell lines in vitro (Figure 2F‐I). Then, subcutaneous xenograft tumour models were established using SK‐LMS‐1 cells with or without *NT5DC2* inhibition. Results showed that tumour weights of the sh*NT5DC2* group were significantly lower than that of the scramble control group (Figure 2J,K).

### 3.3 NT5DC2 interacts with unpalmitoylated TEAD4 and reduces its ubiquitin‐mediated degradation

**FIGURE 3:**
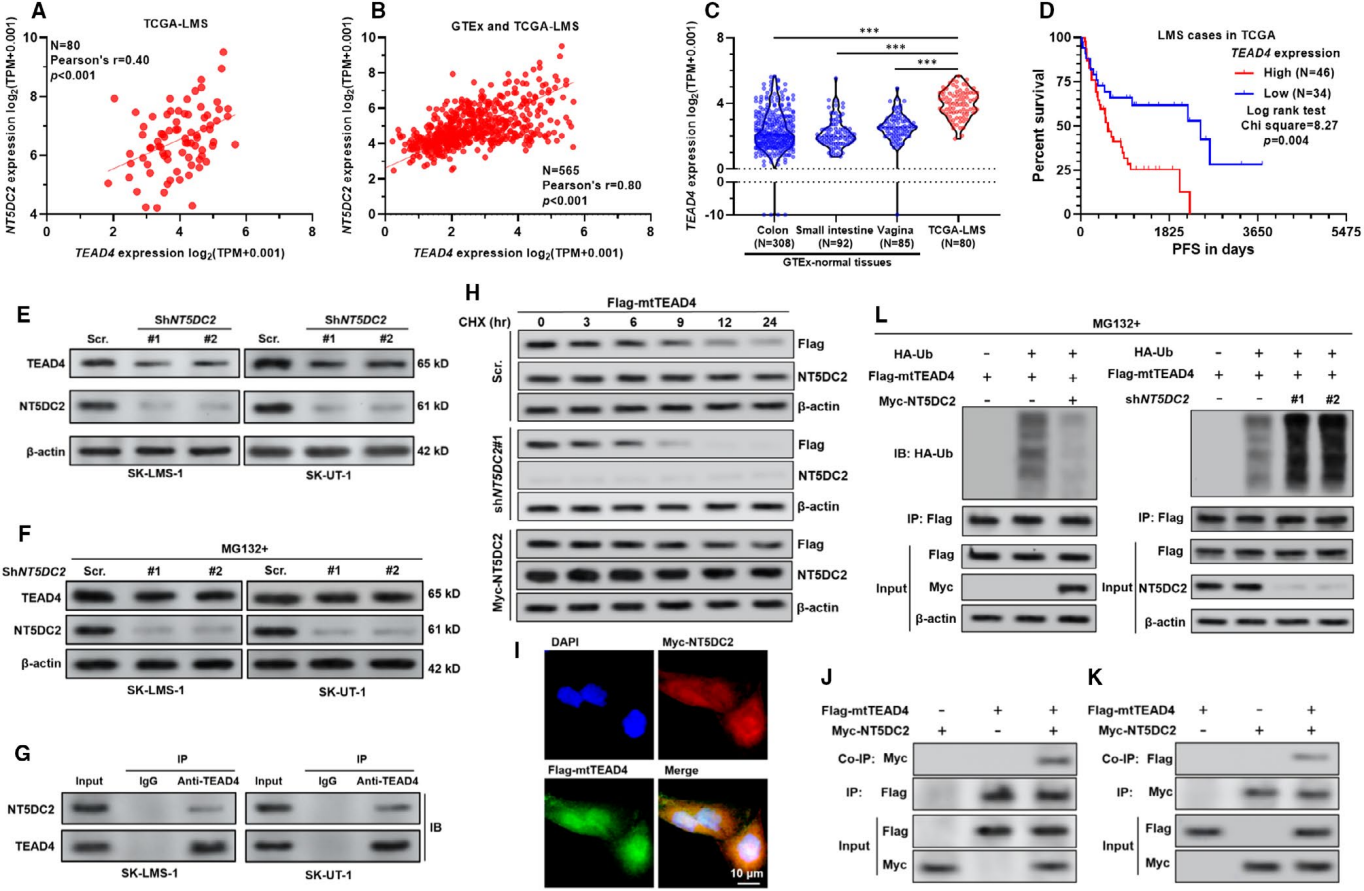
NT5DC2 interacts with TEAD4 and reduces its ubiquitin‐mediated degradation. A and B, Correlation analysis between the expression of *NT5DC2* and *TEAD4* in 80 LMS cases in TCGA (A) and in combined LMS cases and normal smooth muscle tissues (colon, small intestine and vagina) in GTEx (B). C, Comparison of *TEAD4* expression in representative normal smooth muscle tissues, including colon (N = 308), small intestine (N = 92) and vagina (N = 85) from GTEx project and LMS tissues (N = 80). D, K‐M survival analysis of PFS in 80 LMS cases in TCGA. Patients were separated into two groups according to the best cut‐off of *TEAD4* expression. E, Western blot analysis showing the relative *TEAD4* expression in ST‐LMS‐1 and SK‐UT‐1 cells 48 h after infection of lentivirus carrying *NT5DC2* shRNA or scrambled control. F, SK‐LMS‐1 and SK‐UT‐1 cells infected with *NT5DC2* shRNA or scramble control were treated with MG132 (10 μmol/L, 6 h). The expression levels of TEAD4 and NT5DC2 were assessed by western blot assay. G, Co‐IP assays were performed to check the interaction between endogenous NT5DC2 and TEAD4 in SK‐LMS‐1 (left) and SK‐UT‐1 (right) cells. IgG was used as a control. H, Cycloheximide pulse‐chase assay was performed in SK‐LMS‐1 cells with mtTEAD4 overexpression alone or in combination with *NT5DC2* knockdown or overexpression. 36 h after lentiviral infection, cells were treated with 10 μmol/L CHX for the indicated time, followed by western blot analysis. I, Co‐localization of NT5DC2 (red) and mtTEAD4 (green) in SK‐LMS‐1 cells, by immunofluorescent staining. J and K, SK‐LMS‐1 cells were coinfected with Flag‐mtTEAD4 and myc‐NT5DC2 expression lentiviruses. The interaction between NT5DC2 and mtTEAD4 complexes were co‐immunoprecipitated with anti‐Flag (J) or anti‐Myc (K) antibodies. L, SK‐LMS‐1 cells were coinfected with the indicated lentiviruses (Flag‐mtTEAD4 and Myc‐NT5DC2 or sh*NT5DC2*) for 36 h, followed by treatment with MG132 (10 μmol/L, 6 h). Then, cell lysates were immunoprecipitated with an anti‐Flag antibody. Ubiquitinated mtTEAD4 was detected by western blotting with an anti‐HA antibody. *P <.05, **P <.01, ***P <.001

Using RNA‐seq data in TCGA‐LMS (N = 80), we observed a moderate positive correlation (Pearson's *R* = .40) between *NT5DC2* and *TEAD4* expression (Figure 3A). If we added the normal smooth muscle cases from colon, small intestine and vagina in GTEx, the correlation *R*‐value drastically increased to .80 (Figure 3B). Like *NT5DC2*, *TEAD4* was also significantly upregulated in LMS cases, compared with the normal smooth muscle groups (Figure 3C). Under the best cut‐off model, high *TEAD4* expression was associated with worse PFS (Figure 3D).

After *NT5DC2* inhibition, both SK‐LMS‐1 and SK‐UT‐1 cells had downregulated *TEAD4* expression at the mRNA and protein levels (Figure S1A,B and Figure 3E,F). One previous study reported that depalmitoylation of the TEAD4 protein triggers degradation via the ubiquitin‐proteasome pathway.^11^ Since NT5DC2 might act as an inhibitor of ubiquitin‐mediated degradation,^3^, ^5^ we explored whether NT5DC2 inhibits the ubiquitination and degradation of unpalmitoylated TEAD4. Co‐IP assay confirmed that endogenous NT5DC2 was co‐immunoprecipitated by anti‐TEAD4 in SK‐LMS‐1 and SK‐UT‐1 cells (Figure 3G). Cycloheximide pulse‐chase assay was performed in SK‐LMS‐1 cells (Figure 3H). Silencing of *NT5DC2* decreased the stability of unpalmitoylated TEAD4, while *NT5DC2* overexpression increased the half‐life of the protein (Figure 3H).

To validate the interaction between NT5DC2 and unpalmitoylated TEAD4, we co‐expressed myc‐NT5DC2 and Flag‐mtTEAD4 in SK‐LMS‐1 cells. IF staining indicated that mtTEAD4 had both nuclear and cytoplasm distribution. NT5DC2 showed co‐localization with mtTEAD4 in the cytoplasmic part (Figure 3I). Co‐IP assay confirmed the interaction between NT5DC2 and mtTEAD4 (Figure 3J,K). To validate the suppressive effect of NT5DC2 on ubiquitin‐mediated degradation of unpalmitoylated TEAD4, we conducted a ubiquitination assay by overexpressing mtTEAD4 alone or in combination with NT5DC2 overexpression or knockdown. In the presence of MG132, *NT5DC2* overexpression significantly decreased polyubiquitylated mtTEAD4 protein (Figure 3L, left), while *NT5DC2* knockdown significantly increased mtTEAD4 polyubiquitylation (Figure 3L, right).

### 3.4 TRIM27 induces K27/48‐linked ubiquitination of unpalmitoylated TEAD4 at Lys278

**FIGURE 4:**
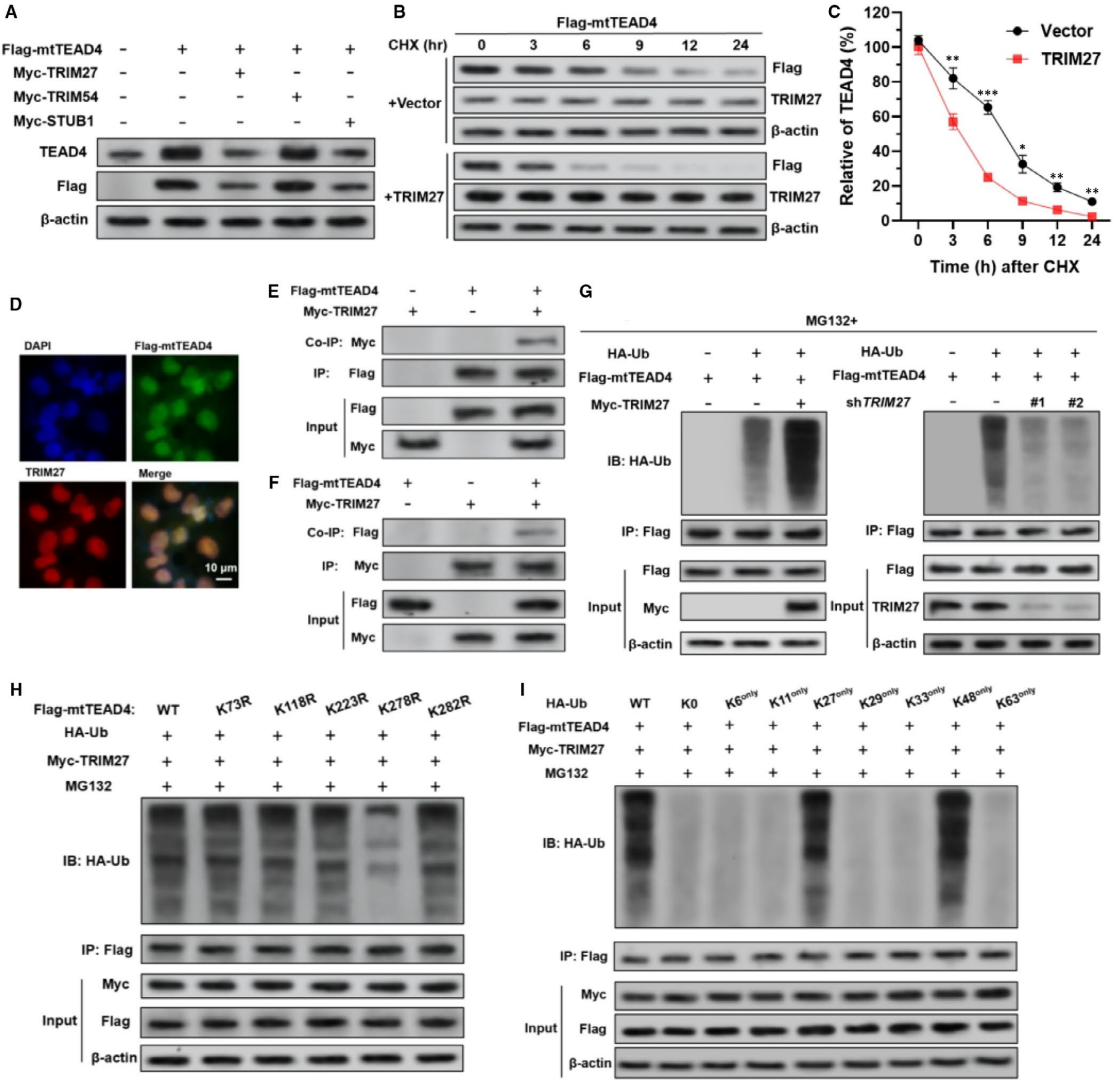
TRIM27 induces K27/48‐linked ubiquitination of unpalmitoylated TEAD4 at Lys278. A, SK‐LMS‐1 cells were coinfected with the indicated lentiviruses for 48 h, for Flag‐mtTEAD4 expression alone or in combination with myc‐TRIM27, myc‐TRIM54 or myc‐STUB1. Then, western blot analysis was conducted to analyse the expression of total TEAD4 and Flag‐mtTEAD4. B and C, Cycloheximide pulse‐chase assay was performed in SK‐LMS‐1 cells with mtTEAD4 overexpression alone or in combination with TRIM27 overexpression. 36 h after lentiviral infection, cells were treated with 10 μmol/L CHX for the indicated time, followed by western blot analysis. The indicated proteins were analysed (B), and the relative mtTEAD4 protein level is illustrated graphically (C). D, Co‐localization of TRIM27 (red) and Flag‐mtTEAD4 (green) in SK‐LMS‐1 cells, by immunofluorescent staining. E and F, SK‐LMS‐1 cells were coinfected with Flag‐mtTEAD4 and Myc‐TRIM27 expression lentiviruses. The interaction between NT5DC2 and mtTEAD4 complexes were co‐immunoprecipitated with anti‐Flag (E) or anti‐Myc (F) antibodies. G, SK‐LMS‐1 cells were coinfected with the indicated lentiviruses (Flag‐mtTEAD4 and Myc‐TRIM27 or sh*TRIM27*) for 36 h, followed by treatment with MG132 (10 μmol/L, 6 h). Then, cell lysates were immunoprecipitated with an anti‐Flag antibody. Ubiquitinated mtTEAD4 was detected by western blotting with an anti‐HA antibody. H, SK‐LMS‐1 cells were infected with wild‐type HA‐Ub, Myc‐TRIM27 and Flag‐mtTEAD4 (WT, K73R mutant, K118R mutant, K223R mutant, K278R mutant and K282R mutant). Co‐IP was conducted as described for panel (G, left). I, SK‐LMS‐1 cells were infected with Myc‐TRIM27 and Flag‐mtTEAD4 and HA‐Ub (WT, K0, K6^only^, K11^only^, K27^only^, K29^only^, K33^only^, K48^only^ and K63^only^). Co‐IP was conducted as described for panel (G, left)

Although ubiquitination mediated TEAD4 degradation has been characterized, E3 ubiquitin ligase is a large class with hundreds of members, the specific E3 ligases linked to TEAD4 have not been fully understood. One previous study reported that STUB1/CHIP is a key enzyme related to this process.^11^ By checking TEAD4 interacting proteins in BioGRID, we found two E3 ubiquitin ligases, TRIM27 and TRIM54 might interact with TEAD4. TRIM27 and STUB1, but not TRIM54 overexpression, significantly reduced mtTEAD4 expression at the protein level but not the mRNA level in SK‐LMS‐1 cells (Figure S1C‐I and Figure 4A), suggesting that TRIM27 might also participate in the degradation of unpalmitoylated TEAD4. Cycloheximide pulse‐chase assay showed that TRIM27 overexpression significantly decreased the half‐life of mtTEAD4 (Figure 4B,C). IF assay indicated co‐localization of TRIM27 and mtTEAD4 in the nuclear part of SK‐LMS‐1 cells (Figure 4D). Co‐IP experiment confirmed the interaction between TRIM27 and mtTEAD4 (Figure 4E,F). In the presence of MG132, TRIM27 overexpression significantly enhanced polyubiquitylation of mtTEAD4 protein (Figure 4G, left), while *TRIM27* knockdown significantly weakened mtTEAD4 polyubiquitylation (Figure 4G, right).

`Seven potential ubiquitination sites at lysine residues were observed in the TEAD4 protein (Figure S2). To identify the site of ubiquitination, we subsequently constructed TEAD4 mutants in which the lysine residues were replaced with arginine. Results indicated that the mutant with K278R had significantly reduced polyubiquitination compared to other mutants (Figure 4H). In addition, TRIM27‐mediated mtTEAD4 polyubiquitination could be detected in the presence of K27‐Ub or K48‐Ub, but not with other Ub mutants (Figure 4I).

### 3.5 *TEAD4* inhibition suppresses LMS cell proliferation in vitro and tumour growth in vivo

**FIGURE 5:**
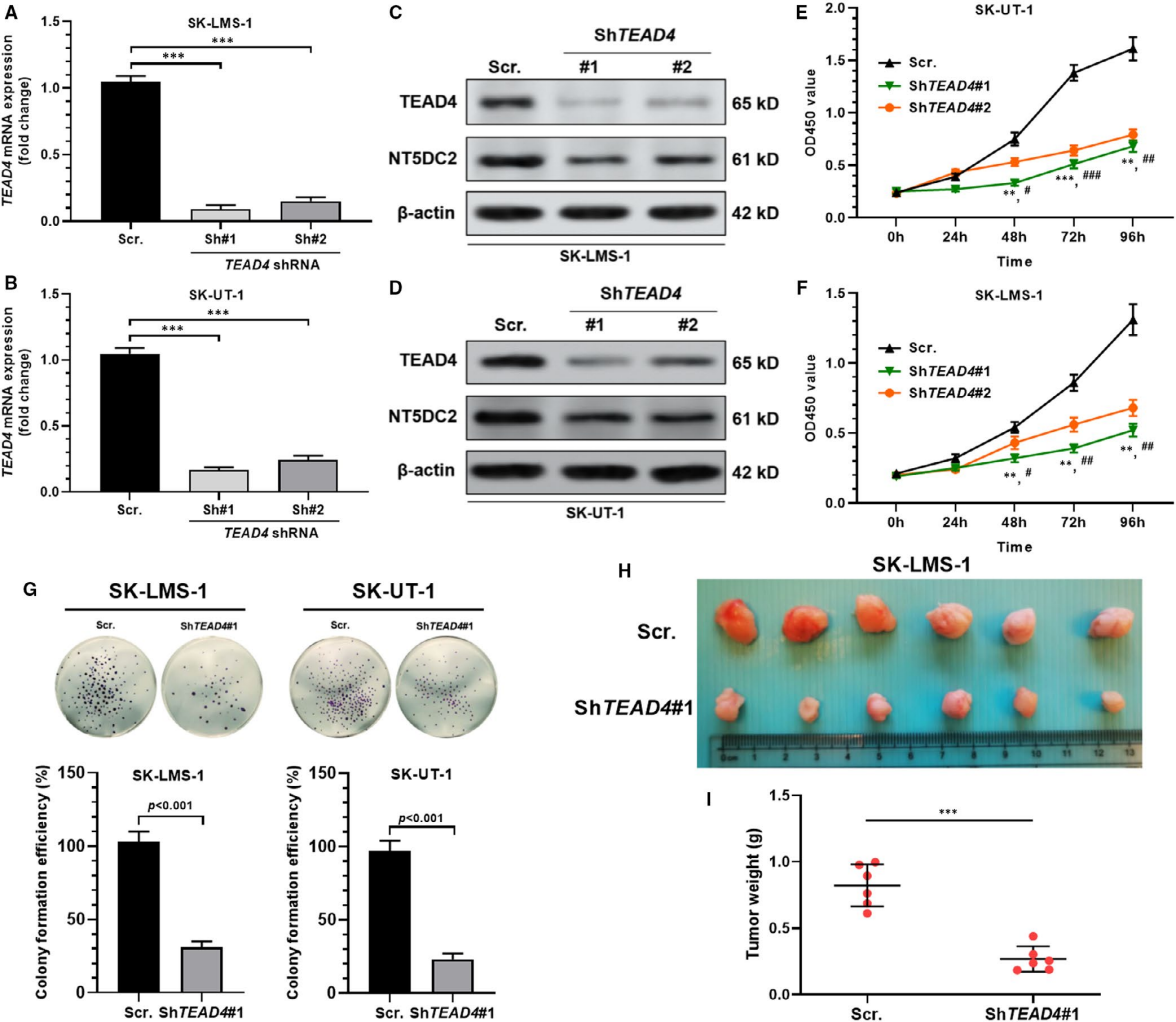
*TEAD4* inhibition suppresses LMS cell proliferation in vitro and tumour growth in vivo. A and B, RT‐qPCR analysis showing the relative *TEAD4* expression in ST‐LMS‐1 (A) and SK‐UT‐1 (B) cells 48 h after infection of lentivirus carrying *TEAD4* shRNA or scrambled control. C and D, Western blot assay of TEAD4 and NT5DC2 expression in ST‐LMS‐1 and SK‐UT‐1 cells 48 h after infection of lentivirus carrying *TEAD4* shRNA or scrambled control. E and F, The proliferation of SK‐LMS‐1 (E) and ST‐UT‐1 (F) cells with or without *TEAD4* knockdown. G, Representative images of colony formation (up) and quantitation or relative colony capability (down) of SK‐LMS‐1 (left) and SK‐UT‐1 (right) cells with or without *TEAD4* knockdown. H and I, SK‐LMS‐1 cells infected with *TEAD4* knockdown (shRNA) or scramble control were subcutaneously implanted into nude mice, and the tumours were harvested 4 weeks later (H). Compared with the controls, *TEAD4* knockdown significantly inhibited tumour growth (I). *Comparison with sh*NT5DC2*#1, ^#^Comparison with sh*NT5DC2*#2, * and ^#^ *P* <.05, ** and ^##^ *P* <.01, *** and ^###^ *P* <.001

Then, we studied the functional role of TEAD4 in LMS cell proliferation. Both SK‐LMS‐1 and SK‐UT‐1 cells were transiently infected with lentiviral‐sh*TEAD4* (Figure 5A‐D). *TEAD4* inhibition remarkably decreased the proliferation and colony‐forming ability of the two cell lines in vitro (Figure 5E‐G). Subcutaneous xenograft tumour models confirmed that knocking down of *TEAD4* substantially reduced tumour growth in vivo (Figure 5H‐I). These findings demonstrated that TEAD4 might also act as a carcinogenetic driver in LMS.

### 3.6 *TEAD4* transcriptionally activates *NT5DC2* expression in LMS

**FIGURE 6:**
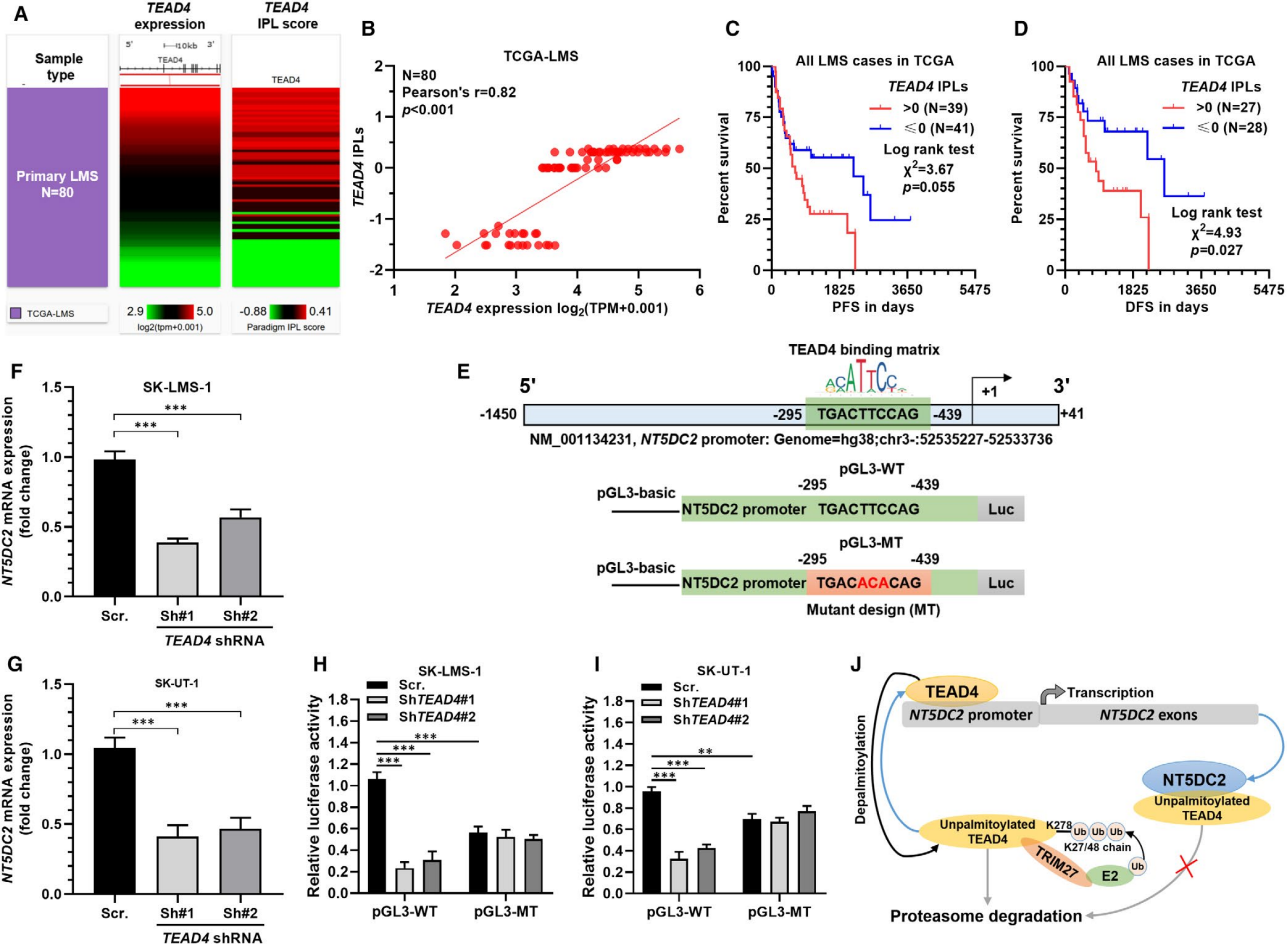
*TEAD4* transcriptionally activates *NT5DC2* expression in LMS. A and B, Heatmap (A) and plot chart (B) showing the correlation between TEAD4 expression and TEAD4 IPLs in 80 LMS cases in TCGA. C and D, K‐M survival analysis of PFS (C) and DFS (D) in 80 LMS cases in TCGA. Patients were separated into two groups according to the activating status of the TEAD4 pathway. E, Predicted TEAD4 binding site in the promoter region of *NT5DC2*. F and G, RT‐qPCR analysis showing the relative *NT5DC2* expression in ST‐LMS‐1 (F) and SK‐UT‐1 (G) cells 48 h after infection of lentivirus carrying *TEAD4* shRNA or scramble control. H and I, Dual‐luciferase assay of comparing the activity of pGL3 plasmids carrying wild‐type or mutant *NT5DC2* promoter sequences in ST‐LMS‐1 (H) and SK‐UT‐1 (I) cells with or without *TEAD4* inhibition. J, A schematic image showing the possible positive feedback loop between NT5DC2 and TEAD4 in LMS tumour cells

Interestingly, by suppressing *TEAD4* expression, we also observed downregulated NT5DC2 expression at the protein level (Figure 5C). This finding implied that there might be mutual regulation between TEAD4 and NT5DC2. As a transcriptional factor, TEAD4 can activate gene expression via promoter binding.^18^ We then checked the IPLs for TEAD4. Among the 80 LMS cases, *TEAD4* expression was positively correlated with TEAD4 IPLs (Figure 6A,B). 39 cases who had TEAD4 IPLs > 0 (activated pathway) tended to have worse PFS, compared to the cases with TEAD4 IPL ≤ 0 (inactivated pathway) (*P* = .055, Figure 6C). Among 55 cases with DFS data, TEAD4 IPLs > 0 group had significantly shorter DFS (*P* = .027, Figure 6D). These findings suggested that the activation of the *TEAD4* downstream pathway is common in LMS and might contribute to disease progression.

We performed bioinformatic screening of the possible downstream targets (N = 27) of the TEAD/YAP complex summarized in one previous study.^19^ Results showed that among the genes with known regulations in different tissues, only *FOXM1* had a moderate positive correlation with *TEAD4*, while 7 other genes had a weakly positive correlation with *TEAD4* (Figure S3A). Suppressing endogenous *TEAD4* or *NT5DC2* significantly decreased *FOXM1* expression at the mRNA level (Figure S3B‐E).

By scanning the promoter region of *NT5DC2*, we observed a high potential TEAD4 binding site (Figure 6E, top). *TEAD4* inhibition resulted in significantly downregulated *NT5DC2* mRNA expression in both SK‐LMS‐1 and SK‐UT‐1 cells (Figure 6F,G). Then, we generated recombinant luciferase reporter plasmids carrying WT or MT NT5DC2 promoter segments (Figure 6E, bottom). Dual‐luciferase assay confirmed that the plasmids with mutant TEAD4 binding site had substantially lower luciferase expression (Figure 6H,I). The luciferase activity of pGL3‐WT, but not pGL3‐MT was significantly impaired by inhibiting endogenous *TEAD4* (Figure 6H,I). In combination with other findings in the current study, we proposed a positive feedback loop between NT5DC2 and TEAD4 in LMS tumour cells. TEAD4 binds to the *NT5DC2* promoter and activates its transcription, while NT5DC2 has a physical interaction with unpalmitoylated TEAD4, reducing its ubiquitin‐mediated degradation (Figure 6J).

By setting TCGA‐SARC as a whole group, we observed that high *NT5DC2* expression was generally associated with significantly shorter PFS and DSS (Figure S4A,C). In comparison, high *TEAD4* expression was generally linked to significantly shorter PFS (Figure S4B), but not DSS (Figure S4D). However, subgroup analysis in different sarcoma subtypes (with over 30 cases) only found an association between high *NT5DC2* expression and unfavourable PFS in undifferentiated pleomorphic sarcoma/myxofibrosarcoma (UPS/MFS) cases (Figure S4I). No significant association was observed in other subgroup analyses (Figure S4E‐H, J‐L). These findings imply that the tumour‐promoting effects of NT5DC2 and TEAD4 might be quite specific in different sarcoma subtypes.

## 4 DISCUSSION

In this study, we found that *NT5DC2* is aberrantly upregulated in LMS, and its overexpression was associated with unfavourable survival, suggesting that it might serve as a prognostic biomarker in LMS. Several recent studies revealed that NT5DC2 upregulation might enhance the malignant phenotypes of some tumours, such as facilitated cell‐cycle progression, proliferation, EMT, angiogenesis, metastasis and cancer stem cell properties.^3^, ^4^, ^17^ Bioinformatic analysis in this study implied that *NT5DC2* expression was strongly correlated with multiple cell‐cycle related genes in TCGA‐LMS cases. Findings in this study showed that deletion of *NT5DC2* significantly reduced the expression of cyclin B1, cyclin A2, cyclin E1 and CDK1 and increased G1 phase arrest in LMS cell lines. Subsequent cellular studies confirmed that inhibiting endogenous *NT5DC2* reduced LMS cell proliferation both in vitro and in vivo.

Functionally, NT5DC2 can act as a stabilizer of some oncoproteins by reducing ubiquitin‐mediated degradation, including fyn in glioma ^3^ and EGFR in HCC.^5^ In this study, we confirmed the physical interaction between NT5DC2 and unpalmitoylated TEAD4 and this association reduced TEAD4 degradation via the ubiquitin‐proteasome pathway. Elevated *TEAD4* expression was observed in multiple tumour tissues. Its expression was highly correlated with clinicopathological parameters and worse prognosis.^20^, ^21^, ^22^ In sarcoma, TAZ and YAP, two transcription co‐activators, can bind to TEAD and enhance the oncogenic effect of the complex.^23^ TEAD‐YAP/TAZ complex has been demonstrated as a powerful enhancer of tumour cell proliferation, due to its transcriptional effects on the expression of multiple tumour‐promoting genes.^19^, ^24^ Although palmitoylation is dispensable for the binding of TEAD4 with YAP/TAZ, it is important for TEAD4 stability.^11^, ^25^ After depalmitoylation, unpalmitoylated TEAD4 can be degraded via the ubiquitin‐proteasome pathway.^11^ STUB1 has been demonstrated as an E3 ubiquitin ligase involved in this process.^11^ However, the type of polyubiquitination and the ubiquitination sites at lysine residues TEAD4 protein have not been identified. In this study, we further demonstrated that TRIM27 is a novel E3 ubiquitin ligase that induces K27/48‐linked ubiquitination of unpalmitoylated TEAD4 at Lys278.

TAZ and YAP are highly expressed and constitutively activated in SK‐LMS‐1 cells.^23^ It was showed previously that the TEAD4‐YAP/TAZ complex can be disrupted pharmacologically by verteporfin, which is a heme analogue.^26^ SK‐LMS‐1 cells treated verteporfin had significantly decreased colony formation in soft agar.^23^ Our cellular and animal studies showed that *TEAD4* inhibition significantly suppressed LMS cell growth both in vitro and in vivo. Using IPL data in TCGA‐LMS, we confirmed that the TEAD4 pathway was activated in around 50% (N = 39) of LMS cases. Our bioinformatic screening of 27 known downstream targets (N = 27) of TEAD/YAP complex indicated that only *FOXM1* had a moderate positive correlation with *TEAD4* in 80 LMS cases in TCGA. The tumour‐promoting effects of FOXM1 have widely been reported, including LMS.^27^, ^28^ Our following RT‐qPCR assay confirmed the regulatory effect of TEAD4 and NT5DC2 on *FOXM1* transcription. This finding suggested that the transcriptional regulation of TEAD4 is tissue/tumour‐specific. By applying dual‐luciferase assay, we demonstrated that TEAD4 could bind to the NT5DC2 promoter and activate its transcription. Therefore, TEAD4 might act as one of the dominant oncoproteins in LMS.

This study also has some limitations. Besides ubiquitin‐mediated degradation, we also observed that NT5DC2 inhibition reduced *TEAD4* expression at the mRNA level. However, the underlying molecular mechanism was not identified in the current study. Although we confirmed a positive feedback loop between NT5DC2 and TEAD4, their downstream effectors in LMS were not well characterized. These issues are supposed to be answered in our future studies.

In conclusion, this study revealed a novel NT5DC2‐TEAD4 positive feedback loop, in which TEAD4 binds to the *NT5DC2* promoter and activates its transcription, while NT5DC2 has a physical interaction with unpalmitoylated TEAD4, reducing its ubiquitin‐mediated degradation. This feedback loop helps to explain the highly activated TEAD4 downstream signalling pathways in LMS and might serve as a potential therapeutic target.

## CONFLICT OF INTEREST

The authors confirm that there are no conflicts of interest.

## AUTHOR CONTRIBUTIONS

**Bowen Hu:** Formal analysis (equal); Methodology (equal); Software (equal); Validation (equal); Visualization (equal); Writing‐review & editing (equal). **Shijie Zhou:** Formal analysis (equal); Validation (equal); Writing‐original draft (equal). **Xuefeng Hu:** Methodology (equal); Resources (equal); Writing‐original draft (equal); Writing‐review & editing (equal). **Hua Zhang:** Formal analysis (equal); Validation (equal); Writing‐review & editing (equal). **Xiaorong Lan:** Formal analysis (equal); Visualization (equal). **Mei Li:** Investigation (equal); Supervision (equal); Writing‐original draft (equal); Writing‐review & editing (equal). **Yunbing Wang:** Project administration (equal); Writing‐review & editing (equal). **Qinsheng Hu:** Conceptualization (lead); Data curation (equal); Investigation (equal); Methodology (equal); Software (equal); Supervision (equal); Validation (equal); Writing‐original draft (lead); Writing‐review & editing (equal).

## Supporting information

Fig S1

Fig S2

Fig S3

Fig S4

Table S1

## Data Availability

All data used in this study were included in the manuscript and Supporting Information.
