# Supplementary figures and images for: NT5DC2 promotes leiomyosarcoma tumour cell growth via stabilizing unpalmitoylated TEAD4 and generating a positive feedback loop
Source: J Cell Mol Med. 2021 May 16;25(13):5976–87. doi: 10.1111/jcmm.16409 (PMC8366447; doi:10.1111/jcmm.16409)

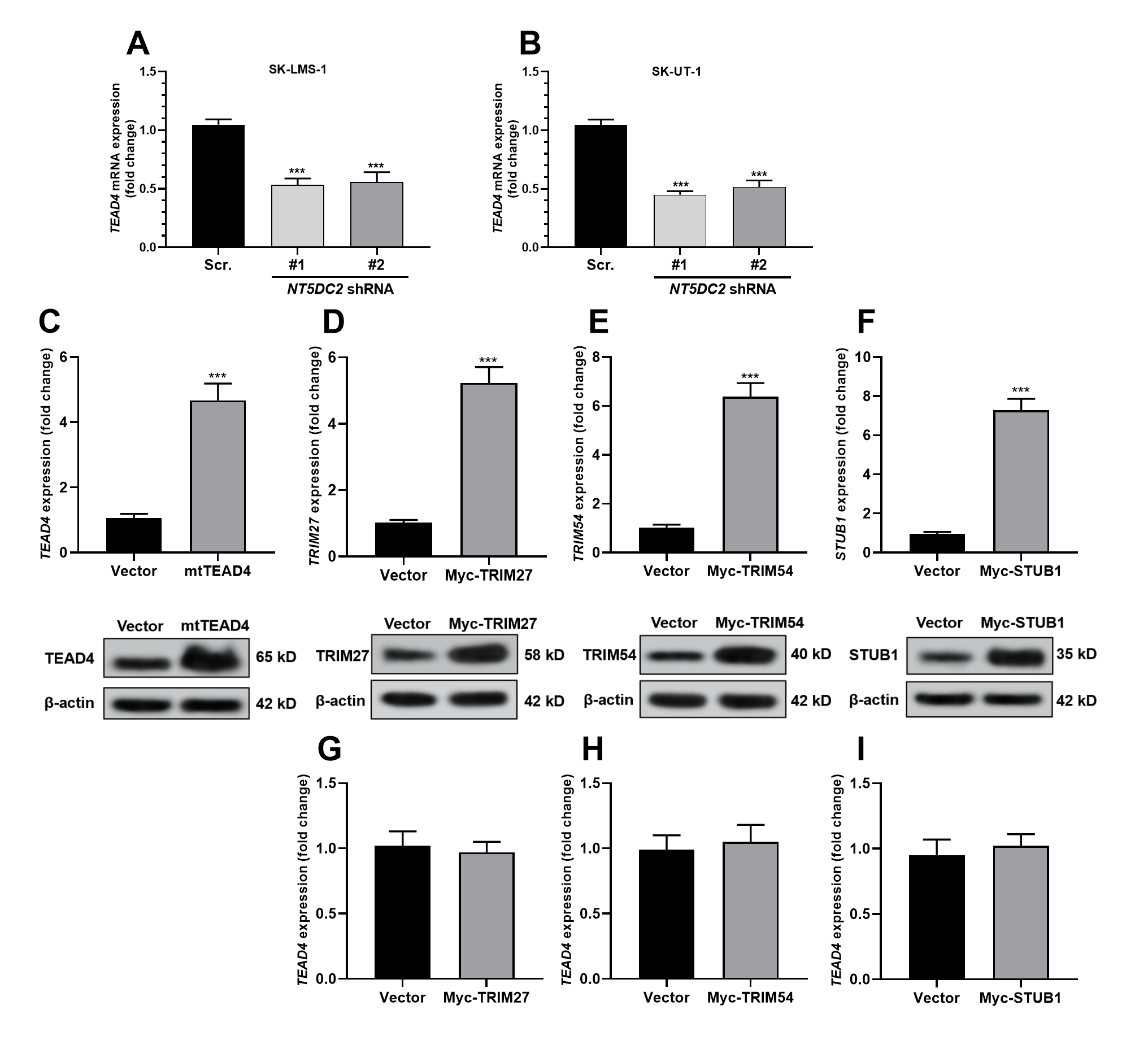

Supplement: Supplementary file 1 — Fig S1 [file JCMM-25-5976-s001.tif]

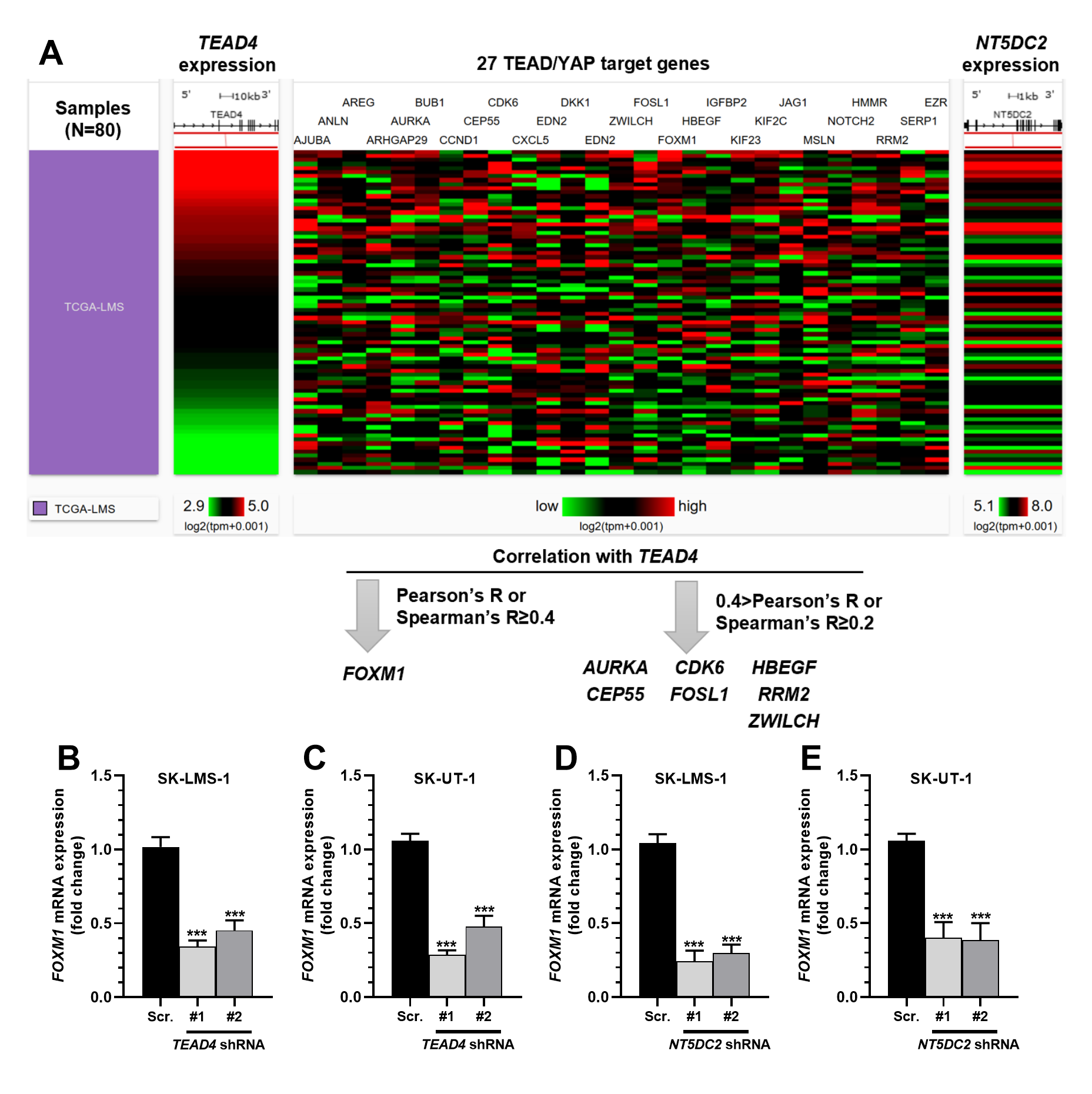

Supplement: Supplementary file 3 — Fig S3 [file JCMM-25-5976-s002.tif]

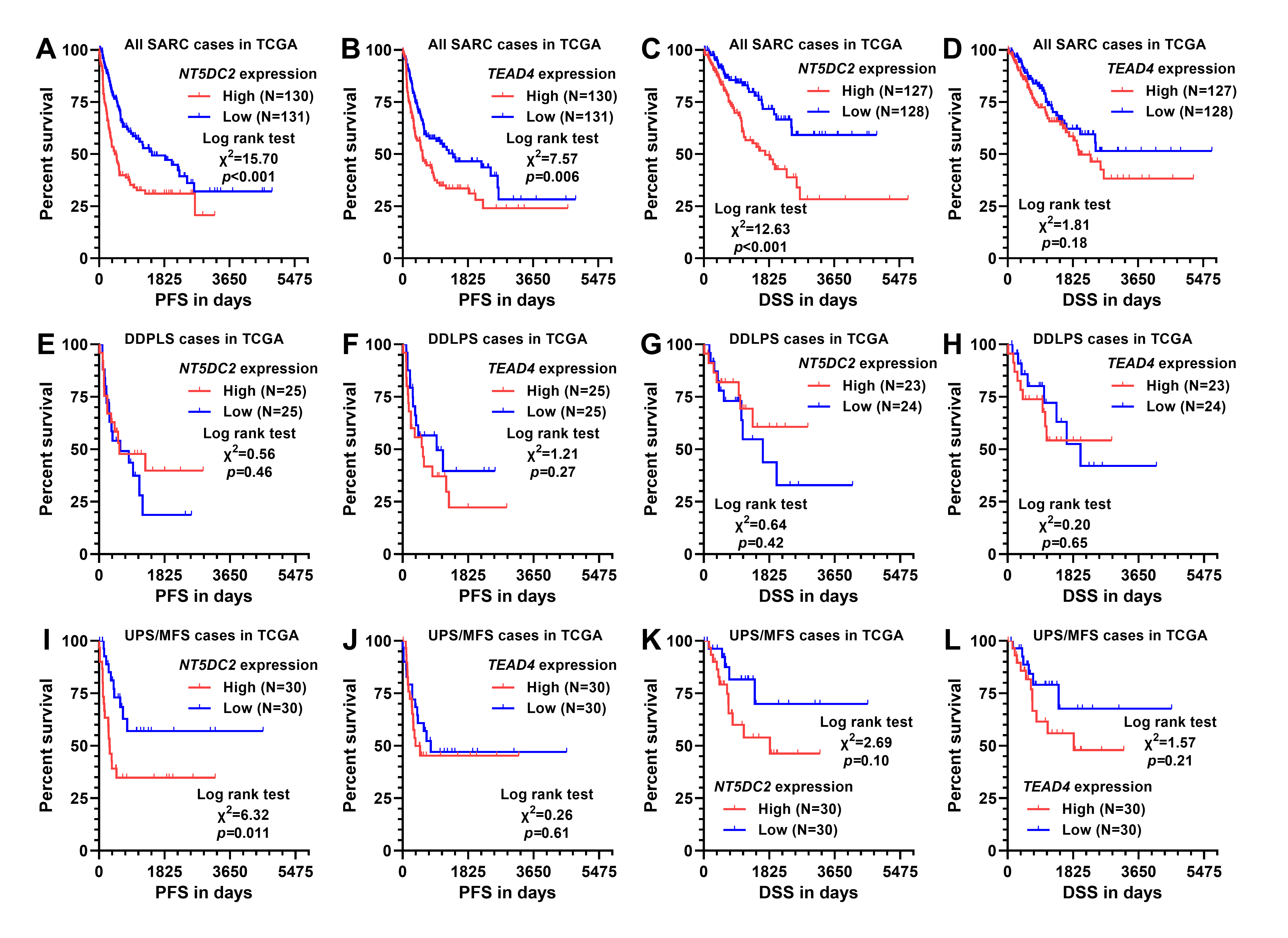

Supplement: Supplementary file 4 — Fig S4 [file JCMM-25-5976-s004.tif]
