# Supplementary material for: NT5DC2 promotes leiomyosarcoma tumour cell growth via stabilizing unpalmitoylated TEAD4 and generating a positive feedback loop
Source: J Cell Mol Med. 2021 May 16;25(13):5976–87. doi: 10.1111/jcmm.16409 (PMC8366447; doi:10.1111/jcmm.16409)
Supplement: Supplementary file 2 — Fig S2 [file JCMM-25-5976-s005.docx]

**Supplementary Figure 2. Potential ubiquitination sites at lysine residues in TEAD4 protein**

10 20 30 40 50
MEGTAGTITS NEWSSPTSPE GSTASGGSQA LDKPIDNDAE GVWSPDIEQS
 60 70 80 90 100
FQEALAIYPP CGRRKIILSD EG**K**MYGRNEL IARYIKLRTG KTRTRKQVSS
 110 120 130 140 150
HIQVLARRKA REIQAKL**K**DQ AAKDKALQSM AAMSSAQIIS ATAFHSSMAL
 160 170 180 190 200
ARGPGRPAVS GFWQGALPGQ AGTSHDVKPF SQQTYAVQPP LPLPGFESPA
 210 220 230 240 250
GPAPSPSAPP APPWQGRSVA SS**K**LWMLEFS AFLEQQQDPD TYNKHLFVHI
 260 270 280 290 300
GQSSPSYSDP YLEAVDIRQI YDKFPEK**K**GG L**K**DLFERGPS NAFFLVKFWA
 310 320 330 340 350
DLNTNIEDEG SSFYGVSSQY ESPENMIITC STKVCSFGKQ VVEKVETEYA
 360 370 380 390 400
RYENGHYSYR IHRSPLCEYM INFIHKLKHL PEKYMMNSVL ENFTILQVVT
 410 420 430
NRDTQETLLC IAYVFEVSAS EHGAQHHIYR LVKE
