# Supplementary material for: NT5DC2 promotes leiomyosarcoma tumour cell growth via stabilizing unpalmitoylated TEAD4 and generating a positive feedback loop
Source: J Cell Mol Med. 2021 May 16;25(13):5976–87. doi: 10.1111/jcmm.16409 (PMC8366447; doi:10.1111/jcmm.16409)
Supplement: Supplementary file 5 — Table S1 [file JCMM-25-5976-s003.docx]

**Supplementary Table 1. ShRNA sequences and primers used in this study**

| **Items** | **shRNA (5'-3')** |
| --- | --- |
| **sh*NT5DC2*#1** | GCAGGGAAACCTGTTTGACTT |
| **sh*NT5DC2*#2** | GTGGCCTCCACTATGACATTC |
| **sh*NT5DC2*#3** | CAGAAGGGATTCGGAAGTATG |
| **sh*TEAD4*#1** | GCTGAAACACTTACCCGAGAA |
| **sh*TEAD4#*2** | GATGTTGGAGTTCTCGGCTTT |
| **sh*TRIM27*#1** | GAATTAAGAGAGGCTCAGTTA |
| **sh*TRIM27*#2** | GCCCTACTTCAGTCTGAGTTA |
|  |  |
|  | **qRT-PCR primer (5'-3')** |
| ***NT5DC2,* F** | CTTCTCGCTACCGGAGATGG |
| ***NT5DC2,* R** | CCGTCACGTCCTTGTAGAGAT |
| ***TEAD4,* F** | GAAGGTCTGCTCTTTCGGCAAG |
| ***TEAD4,* R** | GAGGTGCTTGAGCTTGTGGATG |
| ***TRIM27,* F** | AGCCTGATCGCTCAGCTAGAAG |
| ***TRIM27,* R** | GGAGGTGTGATCCAAGGTTCAG |
| ***TRIM54,* F** | GGAGATGTTCTCCAAACCAGTGG |
| ***TRIM54,* R** | CTCCTGAAGACACAGTGGTGGA |
| ***STUB1,* F** | TCAAGGAGCAGGGCAATCGTCT |
| ***STUB1,* R** | GCATCTTCAGGTAGCACAAGGC |
| ***FOXM1, F*** | TCTGCCAATGGCAAGGTCTCCT |
| ***FOXM1,* R** | CTGGATTCGGTCGTTTCTGCTG |
| ***GAPDH,* F** | GTCTCCTCTGACTTCAACAGCG |
| ***GAPDH,* R** | ACCACCCTGTTGCTGTAGCCAA |
